# The expansion of a patient tracer programme to identify and return patients loss to follow up at a large HIV clinic in Trinidad

**DOI:** 10.1186/s12981-021-00341-3

**Published:** 2021-04-23

**Authors:** R. Jeffrey Edwards, Nyla Lyons, Wendy Samaroo-Francis, Leon-Omari Lavia, Isshad John, Selena Todd, Jonathan Edwards, Gregory Boyce

**Affiliations:** 1 Department of Paraclinical Sciences, Faculty of Medical Sciences, University of the West Indies, St Augustine, Trinidad and Tobago; 2 Medical Research Foundation of Trinidad and Tobago, 7 Queen’s Park East, Port of Spain, Trinidad and Tobago

**Keywords:** Patient tracing, HIV, Intervention, Differentiated care

## Abstract

**Background:**

Patients who default from HIV care are usually poorly adherent to antiretroviral treatment which results in suboptimal viral suppression. The study assessed the outcomes of retention in care and viral suppression by expansion of an intervention using two patient tracers to track patients lost to follow up at a large HIV clinic in Trinidad.

**Methods:**

Two Social Workers were trained as patient tracers and hired for 15 months (April 2017–June 2018) to call patients who were lost to follow up for 30 days or more during the period July 2016–May 2018 at the HIV clinic Medical Research Foundation of Trinidad and Tobago.

**Results:**

Over the 15-month period, of the of 2473 patients who missed their scheduled visits for 1 month or more, 261 (10.6%) patients were no longer in active care—89 patients dead, 65 migrated, 55 hospitalized, 33 transferred to another treatment clinic and 19 incarcerated. Of the remaining 2212 patients eligible for tracing, 1869 (84.5%) patients were returned to care, 1278 (68.6%) were virally unsuppressed (viral load > 200 copies/ml) and 1727 (92.4%) were re-initiated on ART. Twelve months after their return, 1341 (71.7%) of 1869 patients were retained in care and 1154 (86.1%) of these were virally suppressed. Multivariate analysis using logistic regression showed that persons were more likely to be virally suppressed if they were employed (OR, 1.39; 95% CI 1.07–1.80), if they had baseline CD4 counts < 200 cells/mm^3^ (OR, 1.71; 95% CI 1.26–2.32) and if they were retained in care at 12 months (OR, 2.48; 95% CI 1.90–3.24). Persons initiated on ART for 4–6 years (OR, 3.09; 95% CI 1.13–8.48,), 7–9 years (OR, 3.97; 95% CI 1.39–11.31), > 10 years (OR, 5.99; 95% CI 1.74–20.64 were more likely to be retained in care.

**Conclusions:**

Patient Tracing is a feasible intervention to identify and resolve the status of patients who are loss to follow up and targeted interventions such as differentiated care models may be important to improve retention in care.

## Background

The use of anti-retroviral therapy (ART) which effectively suppresses the HIV viral load of patients results in reduced HIV transmission, reduced CD4+ T cell depletion and improved reconstitution of the immune system thereby transforming HIV into a chronic, manageable disease [1–4]. Thus lifelong management of patients with HIV infection requires long term retention on ART [5] which may prove quite challenging as the barriers to retention in HIV care should to be identified and addressed so that the individual and public health benefits of viral suppression can be achieved [6–9]. A systematic review of patient retention in antiretroviral programmes describing 39 cohorts of patients showed that 25% of patients were no longer on ART 24 months after initiation of treatment [10]. Patients who are lost to follow up (LTFU) and non-adherent to ART are at increased risk for HIV transmission, HIV disease progression and subsequent mortality [1, 2, 11, 12], thus it is important to bring those patients with missed appointments back into care [13, 14].

The twin island republic of Trinidad and Tobago are the southernmost islands of the Caribbean and comprise a single nation with a population of approximately 1.36 million (2019 mid-year estimate). The first cases of AIDS were reported in 1983 among gay/bisexual men [15] and by 1985, there was a transition to predominantly heterosexual transmission of HIV [16]. Antiretroviral therapy, subsidized by the government, became available in 2002 and there are approximately 11,000 PLHIV in Trinidad & Tobago with an estimated 27% PLHIV unengaged in care/lost to follow up/not actively on ART treatment [17].

The Medical Research Foundation of Trinidad and Tobago (MRFTT), the largest HIV clinic in Trinidad and Tobago and the Southern Caribbean, recently conducted a 6-month pilot which assessed the feasibility of a patient tracing programme for HIV that re-engaged in care those patients who missed their clinic appointments and the data from this study was previously reported [18]. In this pilot programme, two social workers were trained as patient tracers and of 1058 patients who missed their scheduled visits for 1 month or more, 192 were no longer in active care (deceased, incarcerated, hospitalized, migrated or transferred to another clinic) and 866 were eligible for patient tracing [18]. Of the 866 patients, 589 (68%) were successfully contacted and returned to care [18].

The data and outcomes of this pilot study assisted in allocation of programme resources for targeted interventions to reduce lost to follow up and increase patient retention in HIV care through a tailored package of HIV services to better serve the needs patients enrolled in HIV care [19] to include those patients who regularly miss their clinic appointments, youth, non-virally suppressed patients and the prison population. The MRFTT clinic implemented models of Differentiated Service Delivery (DSD) [20] using a client driven approach to increase patient retention in HIV care, ART adherence and viral suppression. For example, the clinic operating hours were extended during the weekdays [18] and Saturday morning sessions targeting patients who regularly miss their clinic appointments and those newly initiated on ART. Patient/Peer Advocates [18] were trained to mentor and assist patients to overcome the barriers to ART adherence and retention during dedicated clinic visits [18]. A once per month prison outreach program was implemented to improve ART retention among persons living with HIV who were incarcerated. The MRFTT also implemented a monthly youth focused clinic targeting young persons living with HIV aged 18–25 years with a package of services to include text message reminders and enhanced psychosocial care to retain youth PLHIV on ART.

With these differentiated care services available for patients attending the clinic, the purpose of this study was to evaluate the outcomes of an expanded intervention using patient tracers to find patients lost to follow up from the Medical Research Foundation HIV clinic, and monitor their retention and viral suppression 12 months after their return to care.

## Methods

This was a retrospective study to track patients who missed their scheduled clinic visit for over 1 month and were deemed lost to follow up at the HIV clinic, Medical Research Foundation of Trinidad and Tobago (MRFTT). As of June 30, 2018, a total of 6486 patients were reported to be enrolled active in care at the HIV clinic. The patient tracing methods at the MRFTT are described elsewhere [18]. The patient tracers (social workers) spent 15 months (April 2017–June 2018), 5 days per week, contacting patients via phone calls who missed their scheduled visits and did not return to the clinic for over 30 days during the period July 2016–May 2018 and these patients were given an appointment to return to care. Once a patient was re-engaged in care, a HIV viral load (VL) was done at that point, the patient would be re-initiated/started on ART and viral loads (VL) were repeated every 3–6 months and the last VL 12 months after re-engagement in care was recorded for the study.

### Inclusion criteria

Any patient attending the HIV clinic who missed their clinic visit for 30 days or more (lost to follow up) during the period July 2016–May 2018.

### Exclusion criteria

Any patient attending the HIV clinic who missed their clinic visit for less than 30 days (missed appointments) during the period July 2016–May 2018.

Any patient attending the HIV clinic who did not miss clinic visits during the period July 2016–May 2018.

The source population was the patients attending the HIV clinic, MRFTT during the period July 2016-May 2018. Generally, one patient tracer would follow up with one particular patient so that a relationship could be established and trust built up. “Treat all” started in September 2017 at the MRFTT and all patients who returned to care were offered or restarted on ART and followed up bi-weekly, then monthly, then 3 monthly and finally every 6 months once they were stable on ART and their HIV viral loads were suppressed.

All study protocols and procedures were reviewed and approved the University of the West Indies, Campus Research Ethics Committee, St. Augustine, Trinidad. De-identified patient data were extracted for analysis and this was cleaned and organised into an excel spreadsheet database which was imported into the Statistical Package for Social Sciences (SPSS version 25). The data were compiled to a flowchart to examine all the patients who returned to the clinic including those patients who were lost to follow up. The sociodemographic characteristics of the patients were examined by descriptive analyses and the outcomes were stratified by selected descriptive features. The analysis consisted of a Pearson's chi-square test in order to determine if a statistically significant relationship existed between selected descriptive features and the 12-month outcomes of retention in care and viral suppression. Multivariate analysis using logistic regression was then carried out on the variables that had a statistically significant association with the outcomes to identify the influence of these variables on the 12-month outcomes of retention in care and viral suppression. Both the start-up and implementation costs of the interventions were assessed.

Retention in care was defined as a patient in care and having at least two documented clinic visits (physician visit or/pharmacy medication pick-up or laboratory tests) separated by ≥ 90 days during a calendar year. Viral suppression was defined as a viral load of less than 200 copies/ml.

## Results

**Fig. 1:**
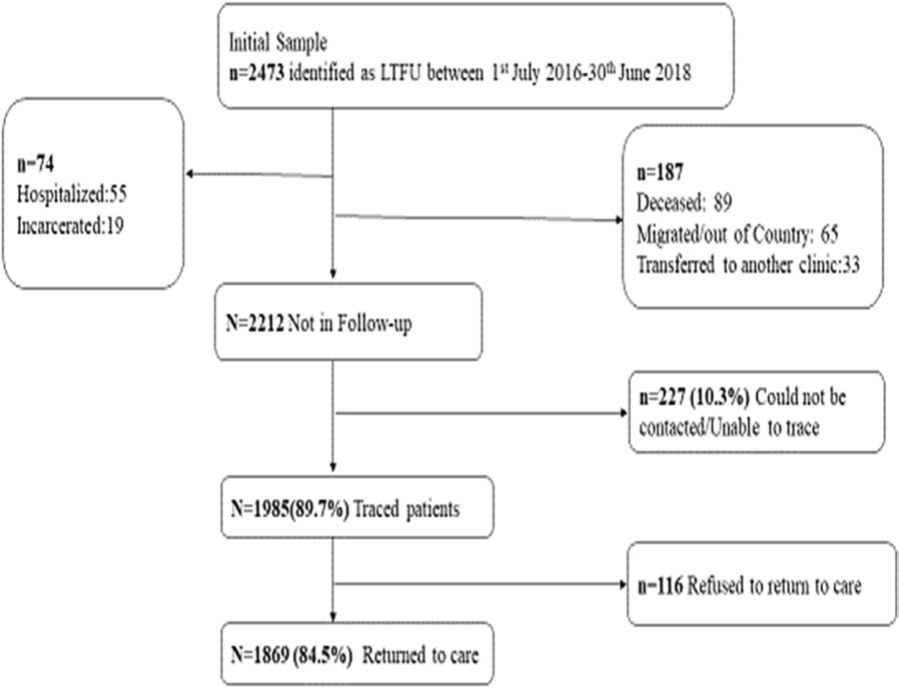
Flow of patient outcomes through the study

Figure 1 shows the flow of patient outcomes through the study over the 15 month period, April 2017–June 2018. Of the sample of 2473 patients who missed their scheduled visits for 1 month or more, it was determined that 261 (10.6%) patients were no longer in active care. These included, 89 patients who were confirmed dead, 65 migrated, 55 hospitalized, 33 transferred to another treatment clinic and 19 incarcerated. Of the remaining 2212 patients eligible for tracing, 227 (10.3%) patients could not be reached up until the end of the study period and 1985 (89.7%) patients were contacted and rescheduled to return to clinic within the same week or their earliest availability. Of those who were reached, 116 did not show up for their rescheduled appointments, thus 1869 (84.5%) of 2212 patients eligible for patient tracing were successfully returned to care (Fig. 1).

Table 1 shows the descriptive characteristics of the 1869 patients returned to care, there were 951 (50.9%) males and 918 (49.1%) females, age range 18–90 years, mean age 43.6 years. There were 21 (1.1%) Female Sex Workers (FSW), 1638 (87.6%) heterosexuals, 221 (11.8%) and 10 (0.6%) self- identified as men who have sex with men (MSM) and lesbians respectively, 985 (71.1%) were employed, 504 (27%) who ever used crack/cocaine/marijuana and 1278 (68.6%) were virally unsuppressed (VL > 200 copies/ml).

**Table 1:** Baseline characteristics of patients LTFU on re-entry to care

Descriptive statistic	Total (n = 1869)
Sex	
Male	951 (50.9%)
Female	918 (49.1%)
Mean age	43.6 years
Age range	18–90 years
Ethnicity (n = 1621)	
African	1168 (72.1%)
East Indian	118 (7.3%)
Mixed/other	335 (20.7%)
Sexual orientation	
Heterosexual	1638 (87.6%)
MSM	221 (11.8%)
Lesbian	10 (0.6%)
Female sex worker (FSW)	21 (1.1%)
Employment status (n = 1386)	
Employed	985 (71.1%)
Unemployed	401 (28.9%)
Drug use (crack/cocaine/marijuana)	
Drug use	504 (27.0%)
Non-drug use	1365 (73.0%)
Viral suppression on re-entry to care (n = 1862)	
Viral Load < 200 copies/ml	584 (31.4%)
Viral Load > 200 copies/ml	1278 (68.6%)
Baseline CD4 count (n = 1859)	
CD4 < 200	620 (33.4%)
CD4 > 200	1239 (66.6%)
Years on ART	
Never initiated ART	75 (4.0%)
≤ 3 years	1027 (54.9%)
4–6 years	396 (21.2%)
7–9 years	308 (16.5%)
≥ 10 years	63 (3.4%)

**Fig. 2:**
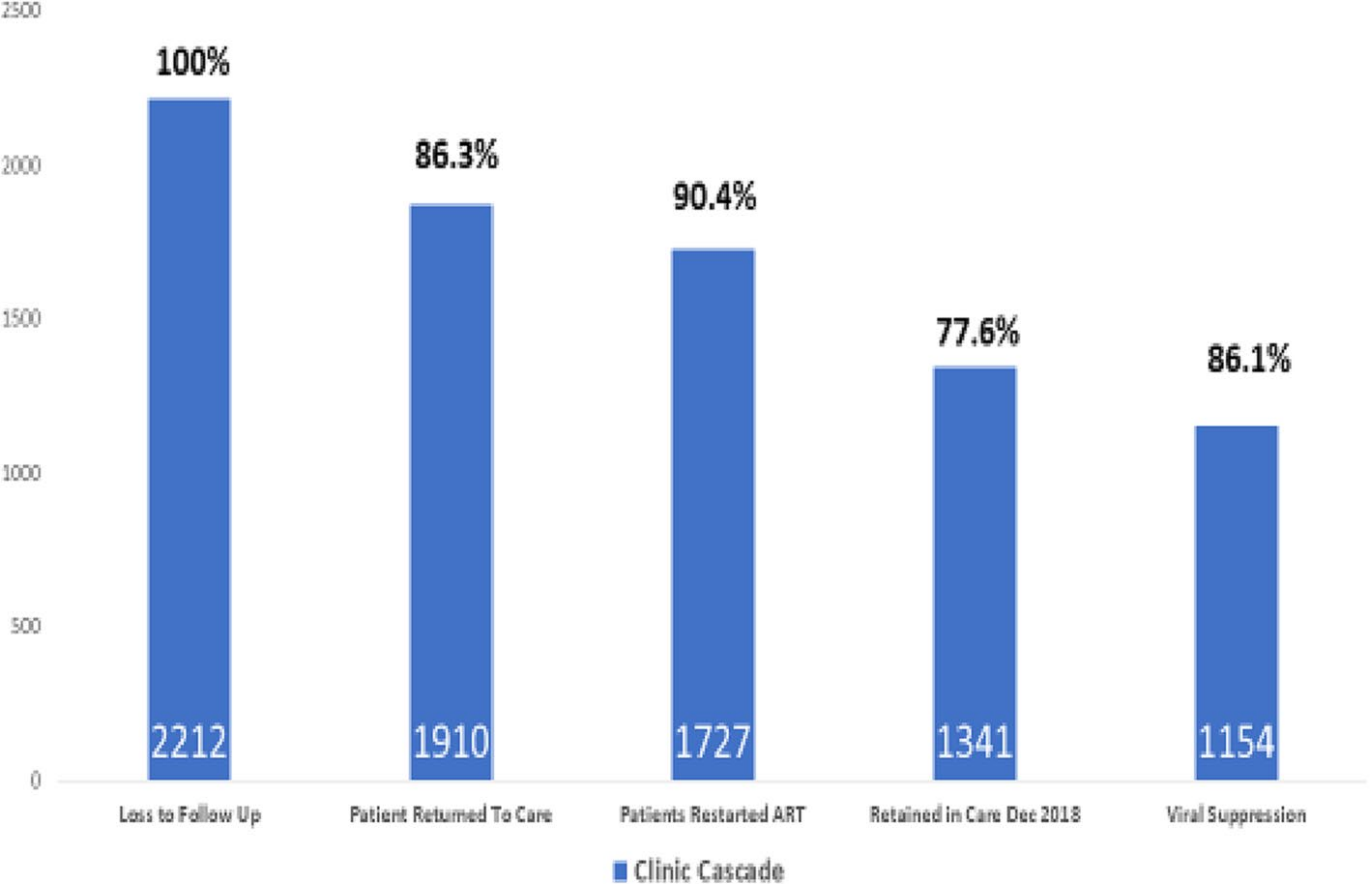
The return to care cascade at the MRFTT, at the end 2018

Figure 2 shows the return to care cascade and tracing outcomes at the MRFTT, of the 1869 patients returned to care, 1727 (92.4%) were re-initiated/started on ART and 12 months after re-engagement in care, 1341 (71.7%) of 1869 patients were retained in care and 1154 (86.1%) of these were virally suppressed (VL < 200 copies/ml).

Table 2 shows the 12-month retention in care and 12-month viral suppression by selected descriptive characteristics. Statistically significant associations for retention in care at 12 months were seen for those persons who were employed (p = 0.01), those with baseline CD4 counts < 200 cells/mm^3^ (p = 0.033) and those who had initiated ART for longer periods of time (p < 0.001). Statistically significant associations for viral load suppression at 12 months were seen for those who were employed (p = 0.001), those who ever used drugs such as marijuana/crack/cocaine (p = 0.007) those with baseline CD4 counts < 200 cells/mm^3^ (p = 0.005), those who had initiated ART for longer periods of time (p < 0.001) and those who were retained in care at 12 months (p < 0.001).

**Table 2:** Selected descriptive characteristics after 12-months of retention in care and viral suppression

Descriptive Statistic	Retention in Care at 12 months N = 1341	OR 95% CI	p value	Viral Suppression at 12 months VL < 200 copies/ml N = 1154	OR 95% CI	p value
Sex		1.13	0.244		0.85	0.09
Male	671 (50.0%)			605 (52.4%)		
Female	670 (50.0%)	0.92–1.38		549 (47.6%)	0.71–1.03	
Ethnicity (n = 1621)			0.065			0.583
African	827 (71.3%)	Ref		706 (71.2%)	Ref	
East Indian	78 (6.7%)	0.829 0.50–1.37		72 (7.3%) 213 (21.5%)	0.910 0.55–1.50	
Mixed/other	255 (22.0%)	1.19 0.84–1.67			1.18 0.85–1.65	
Sexual orientation		0.936	0.669		0.804	0.124
Heterosexual	1178 (87.8%)	0.69–1.27		1022 (88.6%)	0.61–1.06	
MSM	158 (12.2%)			132 (11.4%)		
Employment status (n = 1386)		1.38 1.08–1.77	0.011		1.51 1.19–1.91	0.001
Employed	716 (73.1%)			603 (74.6%)		
Unemployed	264 (26.9%)			205 (25.4%)		
Drug use (crack/cocaine/marijuana)		0.81 0.65–1.02	0.071		0.751 0.61–0.93	0.007
Drug use	346 (75.2%)			286 (24.8%)		
Non-drug use	995 (78.5%)			868 (75.2%)		
Baseline CD4 (n = 1859)		1.27 1.02–1.58	0.033		1.33 1.09–1.63	0.005
CD4 ≤ 200	154 (29.6%)			412 (35.7%)		
CD4 > 200	466 (70.4%)			741 (64.3%)		
Years on ART			< 0.001			< 0.001
Never initiated ART	13 (1.0%)			7 (0.6%)		
≤ 3 years	740 (55.2%)			643 (55.7%)		
4–6 years	296 (22.1%)			276 (23.9%)		
7–9 years	243 (18.1%)			198 (17.2%)		
≥ 10 years	49 (3.7%)			30 (2.6%)		
Retention in care after 12 months					3.18 2.58–3.19	< 0.001
Retained				933 (80.8%)		
Not retained				221 (19.2%)		

Multivariate analysis using logistic regression (Table 3) showed persons were more likely to be retained in care after 12 months if they were employed (OR, 1.41; 95% CI 1.06–1.88) and those who initiated ART for 4–6 years (OR, 3.09; 95% CI 1.13–8.48), 7–9 years (OR, 3.97; 95% CI 1.39–11.31), > 10 years (OR, 5.99; 95% CI 1.74–20.64 compared to those who never initiated ART.

**Table 3:** Results of multivariate logistic regression the variables that had a statistically significant association with the outcomes retention in care and viral suppression

Descriptive statistic	Retained in care N = 1341		Viral suppression VL < 200 copies/ml N = 1154	
	OR 95% CI	95% CI	OR 95% CI	95% CI
Employment status (n = 1386)	1.41	1.06–1.88	1.39	1.07–1.80
Employed				
Unemployed				
Drug use (crack/cocaine/marijuana)			0.823	0.66–1.67
Drug use				
Non-drug use				
Baseline CD4 (n = 1859)	0.886	0.65–1.21	1.71	1.26–2.32
CD4 ≤ 200				
CD4 > 200				
Years on ART				
Never initiated ART	Reference		Reference	
≤ 3 years	2.12	0.80–5.60	1.71	0.38–7.76
4–6 years	3.09	1.13–8.48	1.35	0.29–6.29
7–9 years	3.97	1.39–11.31	1.39	0.29–6.62
≥ 10 years	5.99	1.74–20.64	0.68	0.12–3.76
Retention in care after 12 months			2.48	1.90–3.24
Retained				
Not retained				

Multivariate analysis using logistic regression (Table 3) showed persons were more likely to be virally suppressed at 12 months if they were employed (OR, 1.39; 95% CI 1.07–1.80), if they had baseline CD4 counts ≤ 200 cells/mm^3^ (OR, 1.71; 95% CI 1.26–2.32) and if they were retained in care at 12 months (OR, 2.48; 95% CI 1.90–3.24).

The pilot phase of the programme took place over the 6-month period April-September 2017 and of the 866 patients were eligible for patient tracing, 589 (68%) were successfully contacted and returned to care [18] as compared to 1869 (84.5%) of 2212 patients eligible for patient tracing who were successfully returned to care in the extended programme (p < 0.001). After re-engagement in care for 12 months in the pilot programme, of the 589 patients returned to care, 439 (74.5%) of these were retained in care and 376 (85.6%) of these were virally suppressed as compared to 1341 (71.8%) of 1869 patients who were retained in care (p = 0.89) and 1154 (86.1%) of these were virally suppressed (VL < 200 copies/ml) in the expanded programme (p = 0.93).

Both the start-up and implementation costs of the intervention were assessed (Table 4). The main cost of the intervention was the salary of the two patient tracers, who were employed in pilot phase of programme, initially for 6 months and then in extended programme for 15 months, and the costs of program management which included clerical support, office expenses, services and supplies and bus tickets, a small amount of petty cash ($100 USD/month), snack vouchers and high protein drinks which were given to patients in need (Table 4). The start-up costs i.e. the first month of intervention was $5757 USD per month. The total cost of the intervention in the pilot programme was $28,107.48 USD and in the extended programme was $68,338.20 USD. In pilot programme, 589 patients were returned to care at an estimated cost of $47.72 USD per patient compared to 1869 patients were returned to care in the extended programme (Table 4) at an estimated to be $36.56 USD per patient.

**Table 4:** Estimated costs of returning patients to care at MRFTT April 2017–June 2018

Category	Item	Startup costs		Implementation costs	
		# Units	Initial costs/month one	Recurring costs/over 5 months	Recurring costs/over 14 months
Personnel salaries	Patient tracers	2	$3,582.08	$17,910.40	$50,149.12
	Program management	1	$700	$3500	$9800
Equipment	Laptop	2	$491	$0.00	$.00
	Cell phones	2	$188	$940	$2632
	Workstations	2	$ 296	$0.00	$0.00
Contractual	Training	1	$500	$0.00	$0.00
Subtotal			$5757.08	$22,350.40	$62, 581.12
Total programme costs (startup costs + implementation costs)				$28,107.48 Over 6 months	$68,338.20 Over 15 months

## Discussion

During the 15 month extended programme, two patient tracers working 5 days per week were able to determine the status of 1910 (86.3%) of 2212 patients who were lost to follow up and were eligible for patient tracing. The intervention resulted in returning 1869 (84.5%) of 2212 patients to care as compared to 589 of 866 (68%) patients successfully contacted and returned to care in the 6-month pilot programme (p < 0.001). Thus the effect of the intervention improved over time as more patients were successfully returned to care which may be attributed to the enhanced efficiency of the patient tracing programme as the patient tracers became more proficient at calling patients who missed their clinic appointments and getting them back into care; due to cultivated relationships between the patient tracers and staff from other treatment sites so that patient transfers among sites were more readily ascertained and the MRFTT started a prison programme in January 2018 so that incarcerated patients could be readily identified. In addition, “treat all’ was introduced in September 2017, before this ART was indicated for patients at MRFTT whose CD4 counts fell below 350 cells/mm^3^, so some patients defaulted from clinic as they claimed no treatment was offered to them as their CD4 cell count was “stable”. During the phone calls, the patient tracers explained to the patients the benefits of ART and assured them that once they returned to clinic, ART would be initiated/reinitiated.

Patient tracing programmes have been shown to be effective in determining the status of patients who missed their clinic appointments [14], however there have been mixed results in returning patients to care [21–23]. In a study by Tweya et al. in Malawi, of 1158 patients who missed their clinic visits and were located by tracing, 74% returned to care [21]. Of patients missing clinic appointments in Lusaka, Zambia, Krebs et al. [22] successfully traced 430 (54%) of 789 patients, however only one patient returned to care for every 18 home visits made [22] and in a community based antiretroviral therapy programme in Uganda, of 579 patients sampled for tracing, 61 (12.7%) patients returned to care [23]. In our study, the intervention resulted in 1869 (84.5%) of 2212 patients eligible for tracing being returned to care.

Patients who miss their clinic appointments often have unsuppressed and transmissible viral loads and are generally not included in the ascertainment of the HIV Treatment Cascade of the HIV clinic [24]. Of the 1869 patients returned to care, 1278 (68.6%) were virally unsuppressed, 1727 (92.4%) were re-initiated/started on ART, and after 12 months, 1341 (77.6%) of these were retained in care and 1154 (86.1%) of these were virally suppressed. In the study by Krentz et al. [24], it was found that patients who missed their clinic visits and returned to care had high rates of unsuppressed viral loads (54.5%) especially if the patients were out of care for more than a year [24]. Our study showed that patients who were retained in care after 12 months were more likely to be virally suppressed (OR, 2.48; 95% CI 1.90–3.24) compared to those with suboptimal retention in care and multiple studies have demonstrated this [7, 25, 26]. In addition, Yehia et al. [27] analyzed 35,433 adult patients attending 18 HIV clinics in the USA between 2006–2011 and found that the association between retention in care and viral suppression was more strongly associated with patients with CD4 ≤ 200 cells/mm^3^ [27] which may suggest a more high-quality patient-provider relationship in patients with advanced disease [27, 28]. In our study, it was found that persons were more likely to be virally suppressed at 12 months if they had baseline CD4 counts ≤ 200 cells/mm^3^ (OR, 1.71; 95% CI 1.26–2.32) however the results were not statistically significant for retention in care at 12 months. There are mixed results in patients with CD4 < 200 cells/mm^3^ and subsequent viral suppression as a systematic review by Lailulo et al. [29] done to identify factors associated with ART treatment failure showed that the likelihood of treatment failure was significantly higher in patients with CD4 < 200 cells/mm^3^ (OR, 4.82; 95% CI 2.44–9.52) in resource limited areas perhaps due to HIV related opportunistic infections, increased pill burdens and the potential to develop increased drug toxicity in patients with advanced disease on multiple medications [29].

The International Labour Organization (ILO) conducted a systematic review of 23 studies involving 6674 PLHIV assessing adherence rates and found that persons who were employed at the time of the study were more likely to have achieved optimal ART adherence (OR, 1.39; 95% CI 1.13–1.71) than those who were unemployed [30]. It was found that unemployment, particularly in resource limited settings was linked to the inability of persons to afford related health services, medications and nutritional support which may lead to poor treatment adherence, low viral suppression and ART failure [30]. Our study supports these findings as persons who were employed were more likely to be retained in care at 12 months (OR, 1.41; 95% CI 1.06–1.88) and more likely to be virally suppressed (OR, 1.39; 95% CI 1.07–1.80).

In the study by Rosen and Ketlhapile in South Africa, 20 of 97 patients (21%) who missed clinic visits were returned to care by patient tracing at a cost of $432 USD per patient, which was quite expensive and unsustainable [31]. Table 4 shows the total cost of the intervention in the pilot programme was $28,107.48 USD and in the extended programme was $68,338.20 USD. During the pilot phase, a total of 589 patients were returned to care at an estimated cost of $47.72 USD per patient returned compared to 1869 patients were returned to care in the extended programme at an estimated to be $36.56 USD per patient returned. Thus, once the programme was launched and running efficiently it was cheaper than in the first few months of establishment and the high yield of patients returned to care (84.5%) demonstrated its feasibility and that the programme can be sustainable.

To improve the efficiency of the patient tracing intervention, it is recommended that Trinidad and Tobago (with a population of approximately 1.36 million inhabitants) invest in a National Health Management Information System (HMIS) to link and track HIV cases/patients who transfer to other treatment facilities/clinics which would result in enhanced tracking and monitoring of patients across sites. The inability to track patients who have transferred to other treatment facilities obfuscates inferences about retention in care. Improved reporting of deaths and linking the national death registry to a HIV case surveillance HMIS can also be effective in validating the status of patients not active in care and lost to follow up [31] as well as strengthening active HIV case surveillance in hospitals and in the prisons to assist in identifying patients who are hospitalized and incarcerated.

As in the pilot programme, the three most common barriers to re engagement in care are, (1) patients not remembering their appointments, (2) patients not getting time off from work to attend their clinic appointments, and (3) patients’ fear of being seen attending the HIV clinic [18]. To address these barriers the MRFTT began offering differentiated services (DSD) by extending the clinic/evening hours for patients who were employed and could not get time off from work. The extended/evening clinic provided a patient friendly environment [18] and expedited care to reduce patients waiting times. Patients with financial challenges were provided with bus tickets and meal vouchers to address transportation barriers that impacted their ability to attend clinic appointments.

The ability of the patient tracers to identify and counsel patients and successfully reintegrate them into HIV care further highlights the effectiveness of the Patient Tracing intervention. Patient tracers as trained social workers provided counselling to both treatment experienced and naïve patients assisting them to overcome the barriers to ART reinitiation and/or initiation which is critical component of treat all strategy at the MRFTT. The study found that the number of years since ART initiation increased the likelihood of patients being retained in care after the 12-month period compared to patients who never initiated ART. Patients who were on ART had a higher likelihood of retention in care compared to those not initiated on ART. The results of the analysis demonstrated a higher likelihood for patient retention in care as time since ART initiation increased—hence underscoring the relationship between early ART initiation and retention in care after missed clinic visits. Of the 1869 patients returned to care, 1727 (92.4%) were re-initiated/started on ART, and after 12 months, 1341 (77.6%) of these were retained in care and 1154 (86.1%) of these were virally suppressed. Given these outcomes, the Patient Tracing Programme was critical in reducing treatment interruptions which if left unattended may lead to high levels of viremia with attendant risks of increasing HIV transmission, the potential for drug resistance, the development of opportunistic infections [32], subsequent hospitalization and high costs to the health care system [33].

One of the limitations of the study is the difficulty in tracking patients’ VL over time, given the cross-sectional approach used. This approach may underestimate the VL burden given the changing nature of VL suppression over time [24]. Another limitation is that some patients may have returned to care after the study concluded or may have transferred to other clinics closer to their homes to reduce transportation costs [31], these factors make it challenging to effectively track patient retention in care in care at any given point in time.

Another limitation of the study is the use of a retrospective study design given that only sociodemographic and clinical characteristics data collected as part of routine program reporting were included in the analysis and therefore limiting the comparability of results across similar studies. Also, the data used in the analysis were reported either at baseline or last observed at the time of data extraction. This may also present a bias given that patients may have fallen in and out of care over the period of the analysis–hence making it difficult to measure continuous retention in HIV care.

## Conclusions

Patient Tracing is a feasible and effective intervention to identify and resolve the status of patients who miss clinic appointments, re-engage and retain them in care. In this study, patient tracers as social workers provided enhanced psychosocial support assisting patients to overcome the barriers to ART initiation and/or re-initiation, retention in continuous care, and to achieve and maintain viral suppression. To enhance patient tracing outcomes, targeted interventions are needed to ensure early ART initiation and differentiated care interventions to address client specific barriers as these may improve retention in care and viral suppression. A longitudinal study design will provide additional opportunities to track the long-term effect of differentiated care interventions over time.

## Data Availability

The datasets used and/or analyzed during the study are available from the corresponding author (RJE) on reasonable request.

## Abbreviations

ART: Antiretroviral therapy
DSD: Differentiated service delivery
EPR: Electronic patient records
HMIS: Health management information system
LTFU: Lost to follow up
MRFTT: Medical Research Foundation of Trinidad and Tobago
